# A rare case of severe leptospirosis infection presenting as septic shock in a non-endemic area: a case report and literature review

**DOI:** 10.1186/s12879-023-08367-w

**Published:** 2023-07-31

**Authors:** Junjie Dai, Can Yao, Huaxiang Ling, Binbin Li, Rongchang Chen, Fei Shi

**Affiliations:** 1 The Second Clinical Medical College, Jinan University, Shenzhen, 518020 Guangdong China; 2 Department of Infectious diseases, The Second Clinical Medical College, The First Affiliated Hospital, Shenzhen People’s Hospital, Jinan University, Southern University of Science and Technology), Shenzhen, 518020 Guangdong China; 3 Key Laboratory of Shenzhen Respiratory Diseases, Institute of Shenzhen Respiratory Diseases, The Second Clinical Medical College, The First Affiliated Hospital, Shenzhen People’s Hospital, Jinan University, Southern University of Science and Technology), Shenzhen, 518020 Guangdong China

**Keywords:** Leptospirosis, Thrombocytopenia, Acute febrile illnesses, Differential diagnosis

## Abstract

**Background:**

Leptospirosis is a zoonosis caused by spirochete “genus” leptospira. The clinical presentations of leptospirosis range from an influenza-like presentation of fever and myalgia, to severe forms. Leptospirosis can potentially lead to a misdiagnosis or delay in diagnosis when clinical similarities exist.

**Case presentation:**

A 63-year-old man presented with fever, shock and thrombocytopenia followed by diffuse pulmonary hemorrhage. Peripheral blood Metagenomic Next-generation Sequencing (mNGS) reported Leptospira interrogans. The patient was treated with piperacillin-tazobactam (TZP) plus doxycycline and improved dramatically after 7 days.

**Conclusion:**

We conclude that leptospirosis can potentially lead to a misdiagnosis or delay in diagnosis. Correctly evaluation of thrombocytopenia in acute febrile illnesses facilitates the differential diagnosis of leptospirosis. mNGS can accurately detect Leptospira DNA during the early stage of the infection.

**Supplementary Information:**

The online version contains supplementary material available at 10.1186/s12879-023-08367-w.

## Background

Leptospirosis is a zoonosis caused by spirochete “genus” leptospira [1]. The clinical presentations of leptospirosis range from an influenza-like presentation of fever and myalgia, to severe forms. Severe leptospirosis can present jaundice, renal failure, severe pulmonary hemorrhage syndrome (SPHS), shock, and multiple organ failure [2, 3].With the acceleration of urbanization, leptospirosis is now extremely rare in the cities of China. This epidemiological trend variation can lead to diagnostic errors, in particular when clinical similarities exist. We report a case of severe leptospirosis infection presenting as septic shock in a non-epidemic area.

## Case presentation

**Fig. 1:**
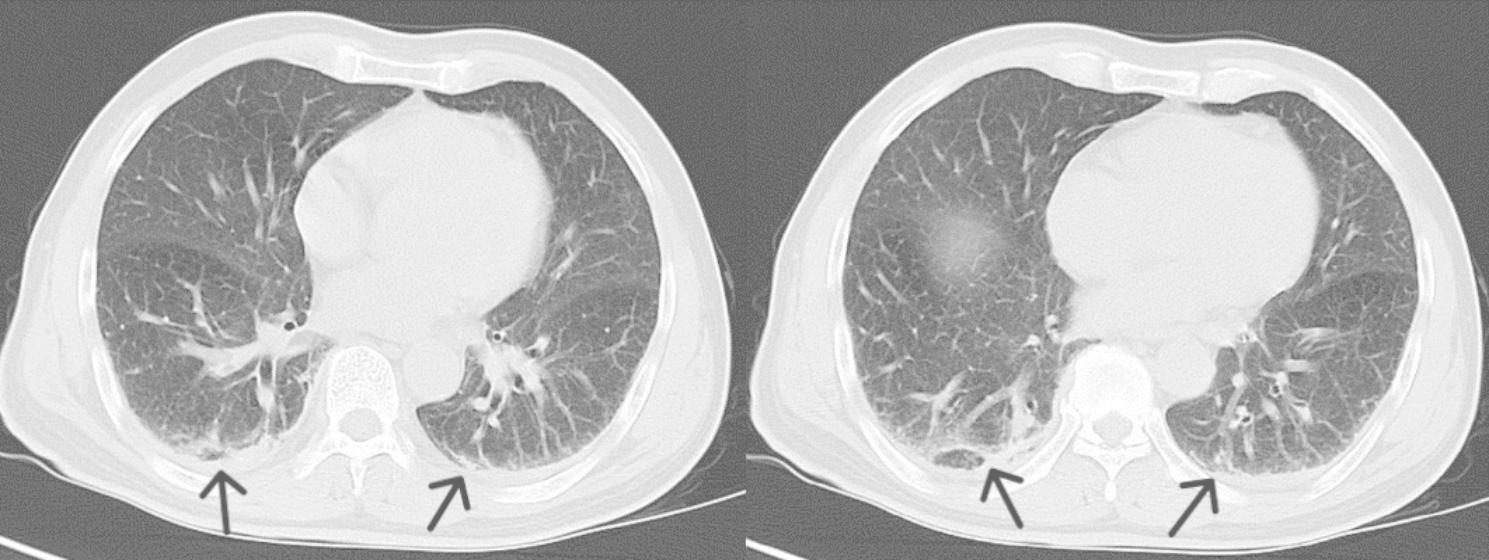
Chest CT of the patient suggesting inflammation of the double lower lungs

**Fig. 2:**
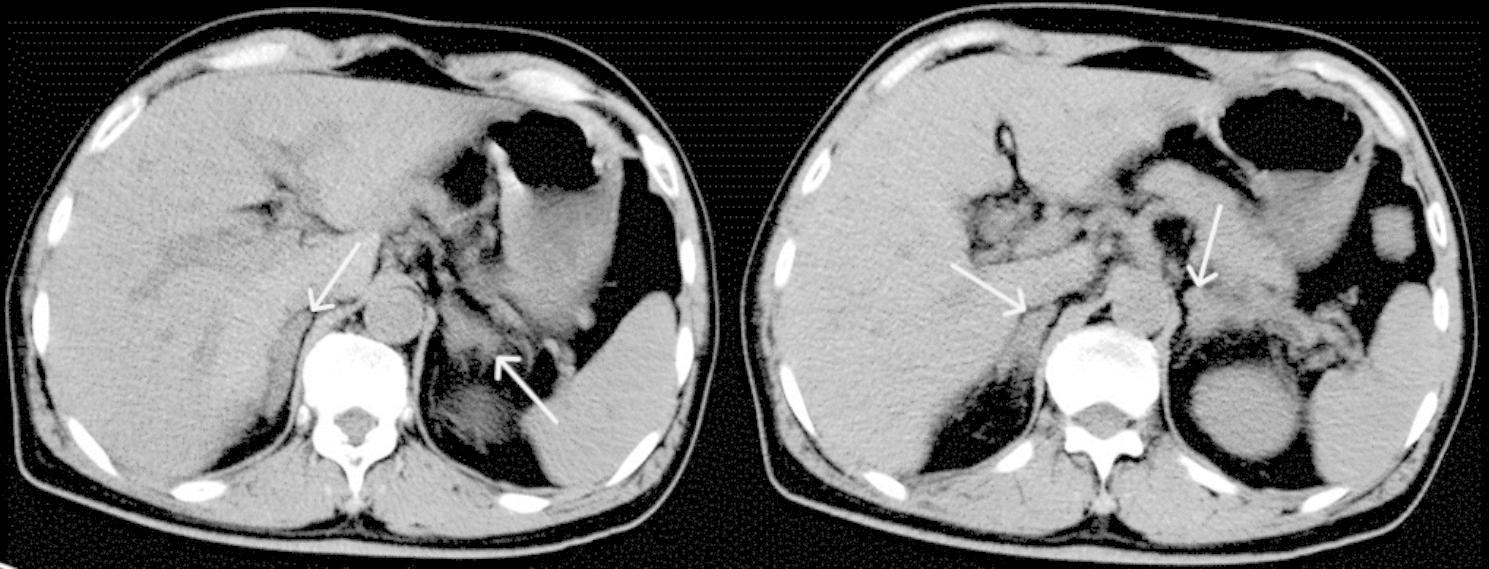
Abdominal CT of the patient showing enlarged adrenal glands

A 63-year-old man presented with a one-day history of shaking chills and high fever. He got wet in the rain 5 days before he came to the hospital, and had a occupational history of septic tank servicer. He had no underlying medical conditions, and denied travel to any areas of endemic. Physical examination revealed blood pressure (80/50 mmHg), a pulse rate of 105 beats per minute and oxygen saturation of 98% on oxygen mask air, and tachypnea with wet rales in both lungs, no conjunctival suffusion and no ictericsclera. Abdominal examination revealed a mild right abdomen tenderness and suspicious positive percussion pain in the kidney area. The rest of the examination, including nervous system, was also unremarkable. Laboratory results (Table 1) showed normal white blood cell (WBC) count 9.14 × 10^9^/L with platelets 7 × 10^9^/L, neutrophils 93.1%, and hemoglobin 85 g/L. Biochemical analysis revealed the increased levels of C-reactive protein (CRP) 209.19 mg/L, procalcitonin (PCT) 68.42ng/mL, interleukin-6 (IL-6) > 5000pg/mL, lactate 5.85mmol/L, creatinine (Cr) 301umol/L, total bilirubin 27.1µmol/L, aspartate transaminase 213.1U/L, alanine transaminase 66U/L, alkaline phosphatase 54U/L, gamma-glutamyl transferase 55U/L, and N-terminal pro b-type natriuretic peptide (NT-proBNP) 3650.97pg/ml (< 125). Arterial blood gas (ABG) analysis showed evidence of type one respiratory failure with mixed respiratory and metabolic acidosis. Urinary analysis revealed the presence of numerous WBC and red blood cells (RBC). The transthoracic echocardiography revealed an ejection fraction of 60%, normal diastolic function and chamber sizes, and color Doppler ultrasonography showed mild mitral, tricuspid and aortic regurgitation. CT examination of chest and abdomen showed inflammation of the double lower lungs, and enlarged adrenal glands (Figs. 1 and 2). The patient was diagnosed with pneumonia, and acute pyelonephritis with septic shock, and intravenous meropenem, fluid resuscitation and vasopressor were prescribed. We implemented the sepsis protocol following the complete hour-1 bundle [4].

**Table 1:** Laboratory results

Day of hospitalization	1	2	3	10	Reference range
Leukocytes (×10^9^/L)	9.14	18.13	15.40	12.42	4–10
Thrombocytes (×10^9^/L)	7	52	62	367	100–300
Neutrophils (%)	93.1	89.4	83	73.9	50–75
Hemoglobin (g/L)	85	76	92	87	120–160
CRP (mg/L)	209.19	236.11	221.68	17.81	< 5.0
PCT (ng/mL)	68.42	105.26	56.67	0.81	< 0.05
IL-6 (pg/mL)	>500	427.3	15.40	11.36	< 7
Lactate (mmol/L)	5.85	4.29	2.67	-	0.7–2.1
Cr (umol/L)	301	296	219	90	44–133
ALT (U/L)	213.1	213.3	123.8	51.9	0–40
AST (U/L)	66	69.4	63.4	39.2	0–45
AP (U/L)	54	-	-	109	15–121
γ-GT (U/L)	55	-	-	125	0–60
Total bilirubin (umol/L)	27.1	37.2	36.2	15,7	1.7–20
NT-proBNP (pg/mL)	3650.97	-	-	-	< 125
pH	7.336	-	-	-	7.35–7.45
PO_2_ (mmHg)	56	-	-	-	83–108
PCO_2_ (mmHg)	27.1	-	-	-	35–45

CRP C-reactive protein, PCT procalcitonin, IL-6 interleukin 6, Cr creatinine, ALT alanine transaminase, AST aspartate transaminase, AP alkaline phosphatase, γ-GT gamma-glutamyl transferase, NT-proBNP N-terminal pro b-type natriuretic peptide

**Fig. 3:**
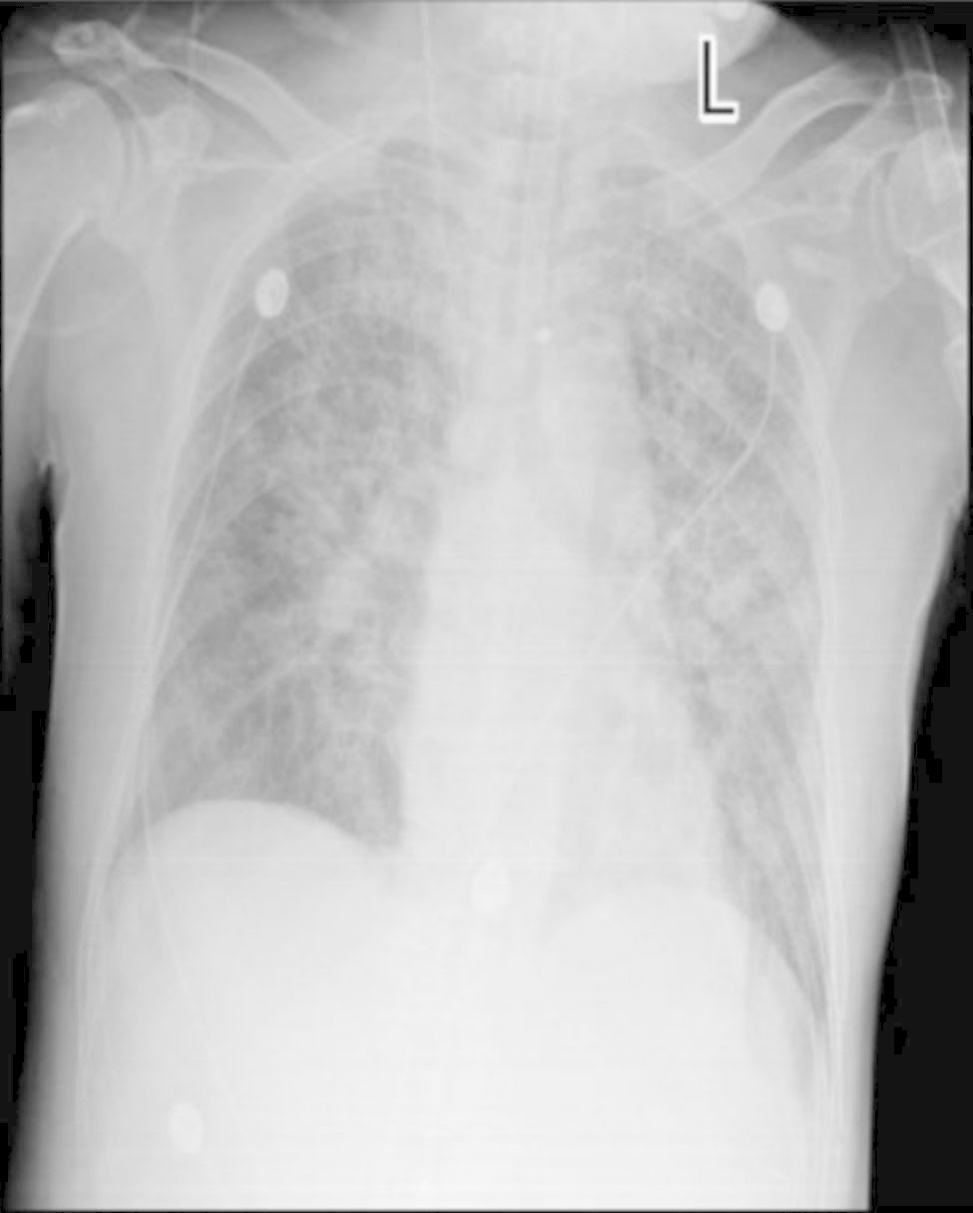
Chest x ray of the patient suggesting diffuse pulmonary hemorrhages

On the second day of admission, the patient developed gradually worsening dyspnea with severe hemoptysis and life-threatening hypoxia. Endotracheal intubation was done and patient was put on a ventilator. Immediately, large clots of blood were extracted from the endotracheal tube (ETT). Bedside chest X-ray demonstrated newly developed bilateral diffuse alveolar shadows suggesting diffuse pulmonary hemorrhages (Fig. 3). We gave the patient a symptomatic treatment with posterior pituitary hormone, hemocoagulase, as well as blood component therapy.

We performed further investigations regarding the etiology of the patient’s condition. Peripheral blood Metagenomic Next-generation Sequencing (mNGS) testing [RDP-seq^®^, Guangzhou Sagene Biotechnology Co., Ltd.], blood and endotracheal aspirate cultures were performed. The results of all cultures were negative after 2 days of incubation. The methods for mNGS were as follows. DNA was extracted from peripheral blood samples using the microbial DNA extraction kit (MAGEN, Guangzhou, China) according to the manufacturer’s protocol. The library was constructed according to the protocol for library construction Kit [Nextera XT^®^, Illumina™, USA]. High-throughput sequencing was performed on the Illumina™ Nextseq 550 DX^®^, sequencing platform (sequencing strategy: SE75), which is an FDA-approved and CE-IVD-certified sequencer. The mNGS sequence results have been uploaded to the NCBI data (accession number: SRR24583305). The mNGS data identified Leptospira interrogans with sequence number 23 and confidence of 99%. The sequence results of identifying Leptospira interrogans were shown in Supplementary material 1. According to the clinical and microbiological findings, the antimicrobial therapy was switched to piperacillin-tazobactam (TZP) plus doxycycline.

Intravenous TZP 4.5 g every 8 h and doxycycline 0.1 g orally every 12 h were administered, and continued for 7 days along with supportive care. Ventilator support was offered for 5 days. Finally, the patient was weaned from the ventilator for successful extubation, and improved dramatically. Normal levels of renal function, cardio-respiratory functions, and hematological parameters were also improved by the end of ten days.

## Discussion and conclusions

Here, we report of a patient who presented with fever, shock and thrombocytopenia followed by diffuse pulmonary hemorrhage caused by leptospirosis. The patient also had elevated levels of CRP, PCT, IL-6, lactate, Cr, urine RBC and WBC. Because acute pyelonephritis often leads to serious conditions including sepsis and septic shock, the patient was diagnosed with acute pyelonephritis with septic shock on the day of hospitalization. However, antibiotic treatment with meropenem was ineffective. To our knowledge, this is not the first case of leptospirosis which went undiagnosed. Similar clinical presentations with thrombocytopenia and shock in acute febrile illnesses often lead to a diagnostic and therapeutic dilemma. Correctly evaluation of thrombocytopenia in acute febrile illnesses plays a crucial role in reducing disease complication and mortality.

Leptospirosis is a worldwide infection of zoonotic origin caused by spirochetes of the genus Leptospira [2]. Leptospirosis transmission usually occurs through direct/indirect contact with urine or tissue of carrier mammals, contaminated water, soil, or vegetation [5]. Portals of entry can be either skin, the respiratory tract or the gastrointestinal tract. Intact skin is an important barrier against leptospiral infection, and skin breakdown is one of the main ways to cause infection [6]. The clinical spectrum of the disease ranges from a mild anicteric leptospirosis manifesting as an influenza-like presentation of fever and myalgia, to multi organ dysfunction including thrombocytopenia, diffuse pulmonary hemorrhage, acute kidney injury, hepatic dysfunction and shock [2, 3, 7]. As a result of its broad range of clinical manifestations that mimic a wide variety of acute infectious diseases, leptospirosis can potentially lead to a misdiagnosis or delay in diagnosis.

The degree of thrombocytopenia in infections has an important value. It can also help in differential diagnosis and clear identification of aetiology of acute febrile illnesses.

Sepsis, zoonotic diseases (including malaria, dengue, scrub typhus, and leptospirosis), and Thrombotic Thrombocytopenic Purpura (TTP) are some of the common causes of fever with thrombocytopenia. Thrombocytopenia occurs in around 18% of patients with sepsis, however, severe thrombocytopenia (platelets < 20,000/mm^3^) is less common in patients with sepsis [8]. Meanwhile, in a cohort of adults with severe malaria in Asia, 95% of patients with severe malaria were thrombocytopenic (platelets < 150,000/mm^3^) and 51% had a platelets < 50,000/mm^[3 [9]]^. Furthermore, acute febrile patients with thrombocytopenia should be considered for the presence of atypical pathogens such as dengue and epidemic hemorrhagic fever infections [10]. Dircio Montes, et al. concluded that one sixth of the patients initially diagnosed with dengue fever were actually leptospirosis [11]. Moreover, scrub typhus is one of the differential diagnoses for fever with thrombocytopenia, and the mainstay of diagnosing scrub typhus, immuno-florescence antibody test, or indirect immuno-peroxidase assay is the gold standard [12]. In our study, the patient’s platelets were only 7 × 10^9^/L on admission, combined with fever, anemia, renal failure, elevated lactate, so the possibility of TTP cannot be excluded. There are some methods that can be employed to identify TTP, such as peripheral blood morphology, bone marrow aspiration, ADAMTS13 activity test, and inhibitor titer test [13]. Therefore, the infectious and non-infectious causes mentioned above should be actively considered in acute febrile patients with thrombocytopenia.

Several diagnostic tests are available for leptospirosis including detection of the pathogen and antibodies. Leptospira culture is an aetiological test with high specificity, and considered as the gold standard test for diagnosis [14, 15]. However, it can take up to about 13 weeks and does not aid the early diagnosis of Leptospira [16].Enzyme-linked immunosorbent assay (ELISA) for detecting IgM, IgG, or both antibody types have been developed to detect specific antibodies in leptospirosis, while the method is prone to false-positive diagnosis [16, 17].As a complementary approach to conventional methods, mNGS is increasingly being applied in clinical laboratories for the diagnosis of leptospirosis, and can accurately detect Leptospira DNA during the early stage of the infection [16, 18]. Early diagnosis of leptospirosis is crucial to provide early and appropriate empiric antibiotics. Therefore, mNGS is recommended when leptospirosis infection is suspected, especially in patients with a history of contacting with contaminated water or animals.

Treatment of severe leptospirosis patients is supportive management, and use of appropriate antibiotics. Penicillin, doxycycline, ceftriaxone, and cefotaxime have been recommended for the treatment of leptospirosis [7, 19]. In the study, the patient received TZP plus doxycycline for 7 days along with supportive care. He improved dramatically, and became asymptomatic.

Overall, Leptospirosis is easily overlooked in cities, and can have serious complications such as septic shock. When a patient presents with unexplained fever, shock, thrombocytopenia, and diffuse pulmonary hemorrhage, mNGS should be considered to identify any special infection including Leptospirosis during the early stage.

## Electronic supplementary material

Below is the link to the electronic supplementary material.

Supplementary Material 1

## Data Availability

All data generated or analysed during this study are included in this published article and its supplementary information files. The mNGS sequence results have been uploaded to the NCBI data (accession number: SRR24583305).

## Abbreviations

mNGS: Metagenomic next-generation sequencing
TZP: piperacillin-tazobactam
CT: Computed tomography
WBC: white blood cell
CRP: C-reactive protein
PCT: procalcitonin
IL-6: interleukin-6
Cr: creatinine
RBC: red blood cells
TTP: Thrombotic Thrombocytopenic Purpura
